# Antepartum Fetal Surveillance and Optimal Timing of Delivery in Diabetic Women: A Narrative Review

**DOI:** 10.3390/jcm13020313

**Published:** 2024-01-05

**Authors:** Alan Braverman-Poyastro, Blanca Vianey Suárez-Rico, Héctor Borboa-Olivares, Salvador Espino y Sosa, Johnatan Torres-Torres, Lidia Arce-Sánchez, Nayeli Martínez-Cruz, Enrique Reyes-Muñoz

**Affiliations:** 1 Community Interventions Research Branch, Instituto Nacional de Perinatología “Isidro Espinosa de los Reyes”, Montes Urales 800, Mexico City 11000, Mexico; alanbraverman24@gmail.com (A.B.-P.);; 2 Facultad de Ciencias de la Salud, Universidad Anáhuac México, Campus Norte, Av. Universidad Anáhuac 46, Huixquilucan 52786, Mexico; 3 Research Direction, Instituto Nacional de Perinatología “Isidro Espinosa de los Reyes”, Montes Urales 800, Mexico City 11000, Mexico; blancasuarezrico@gmail.com; 4 Clinical Research Branch, Instituto Nacional de Perinatología Isidro Espinosa de los Reyes, Mexico City 11000, Mexico; salvadorespino@gmail.com (S.E.y.S.); johnatan.torres@inper.gob.mx (J.T.-T.); 5 Coordination of Endocrinology, Instituto Nacional de Perinatología “Isidro Espinosa de los Reyes”, Montes Urales 800, Mexico City 11000, Mexico; li_arce@yahoo.com.mx (L.A.-S.); nayemc_21@hotmail.com (N.M.-C.); 6 Coordination of Gynecological and Perinatal Endocrinology, Instituto Nacional de Perinatología “Isidro Espinosa de los Reyes”, Montes Urales 800, Mexico City 11000, Mexico

**Keywords:** gestational diabetes mellitus, pregestational diabetes mellitus, antepartum fetal surveillance, fetus, resolution of pregnancy, fetal surveillance tests

## Abstract

Antepartum fetal surveillance (AFS) is essential for pregnant women with diabetes to mitigate the risk of stillbirth. However, there is still no universal consensus on the optimal testing method, testing frequency, and delivery timing. This review aims to comprehensively analyze the evidence concerning AFS and the most advantageous timing for delivery in both gestational and pregestational diabetes mellitus cases. This review’s methodology involved an extensive literature search encompassing international diabetes guidelines and scientific databases, including PubMed, MEDLINE, Google Scholar, and Scopus. The review process meticulously identified and utilized pertinent articles for analysis. Within the scope of this review, a thorough examination revealed five prominent international guidelines predominantly addressing gestational diabetes. These guidelines discuss the utility and timing of fetal well-being assessments and recommendations for optimal pregnancy resolution timing. However, the scarcity of clinical trials directly focused on this subject led to a reliance on observational studies as the basis for most recommendations. Glucose control, maternal comorbidities, and the medical management received are crucial in making decisions regarding AFS and determining the appropriate delivery timing.

## 1. Introduction

Diabetes mellitus (DM), a chronic metabolic disorder experiencing a relentless rise, poses significant challenges in the realm of antepartum care [1,2,3]. According to the International Diabetes Federation (IDF), it is estimated that 21.1 million (16.7%) of live births to women in 2021 had some form of hyperglycemia during pregnancy [4]. Of these, 80.3% were due to gestational diabetes mellitus (GDM), while 10.6% were the result of diabetes detected prior to pregnancy, and 9.1% due to diabetes (including type 1 and type 2) first detected in pregnancy [4]. The prevalence of GDM in Mexico is unknown; however, some studies have reported rates between 10% and 30% for affected Mexican women [5,6,7].

GDM is defined as the presence of diabetes diagnosed in the second or third trimester of pregnancy, which is not overt diabetes prior to pregnancy [8]. On the other hand, pre-gestational diabetes mellitus (PDM) refers to the pre-existing diagnosis of the condition in women who either had the illness before they became pregnant or are diagnosed during pregnancy (meeting criteria for overt diabetes—fasting glucose ≥ 126 mg/dL, random glucose ≥ 200 mg/dL, glycated hemoglobin A1C ≥ 6.5%) [8].

Diverse factors increase the risk of developing diabetes. The American College of Obstetricians and Gynecologists (ACOG) [3] and the American Diabetes Association (ADA) [8] recommend the intentional (selective) screening for diabetes in women with a body mass index (BMI) > 25 and any of the risk factors listed in Table 1, and recommend testing all individuals for undiagnosed diabetes at the first prenatal visit using standard diagnostic criteria [3,8]. 

A recent review that analyzed data from all over the world about the prevalence and modifications to the screening criteria for GDM across all continents showed the lack of a universal consensus on screening and diagnosis criteria for GDM [9].

Most international guidelines recommend screening for GDM in all pregnant women between 24 and 28 weeks of gestation [3,8,9,10], except the National Institute for Health and Care Excellence (NICE) guideline, which recommends screening for GDM only among high-risk-factor women [11] (see Table 2). In the United States of America, according to the ADA, two strategies can be used to diagnose GDM: the one-step 75 g OGTT or the two-step method with a no fasting screen of 50 g, followed by a 100 g OGTT for screened positive patients (see Table 2) [1,9]. This two-step method was also adopted by the ACOG [3,9].

Insulin resistance (IR) and relative pancreatic β-cell dysfunction are central features of GDM [1,16,17]. Consequently, blood glucose is slightly elevated, and this glucose is transported across the placenta to fuel the growth of the fetus. A mild state of insulin resistance also promotes the release of fat stores, increasing blood glucose and free fatty acid (FFA) levels [17]. 

The optimal detection and treatment of GDM reduce maternal and neonatal adverse outcomes and mortality, including large for gestational age (LGA, defined as any fetus or infant weighing more than the 97th percentile) [18,19], macrosomia (defined as an estimated fetal weight or birth weight greater than 4500 g, but a cutoff greater than 4000 g is also frequently used) [19], shoulder dystocia, and preeclampsia [1,3,9,10].

Women with DM in pregnancy are at higher risk of complications such as miscarriage, preeclampsia, preterm birth, congenital malformations, cesarean section, macrosomia, small for gestational age (SGA) and LGA newborns, shoulder dystocia, stillbirth and increased neonatal morbidity and mortality [1,3,11,13,15,20,21,22,23,24].

Fetal surveillance tests (FST), through diverse clinical and technological methods, play a pivotal role in the early detection of fetal health deviations. These tests allow for predicting complications in the baby throughout the pregnancy and after delivery [25]. Since their beginning, the objectives of FSTs have been to identify fetuses at risk of intrauterine death and those prone to developing neurological complications (secondary to chronic intrauterine hypoxia), and to intervene to prevent these adverse outcomes [26,27].

The primary goal of FST is to identify fetuses at risk of adverse outcomes, minimize fetal morbidity, and prevent or reduce perinatal mortality [25,26,27,28,29]. Currently, various international guidelines provide recommendations for fetal monitoring [10,21]. However, their usefulness is controversial, and there is no universal recommendation regarding the different fetal surveillance tests (FST) in DMG and PDM [21]. Similarly, the optimal timing for the resolution of the pregnancy, based on fetal morbidity, has not been standardized in women with DM in pregnancy [10].

The primary aim of this review is to analyze the recommendations of international guidelines and clinical studies assessing AFS testing, such as the non-stress test (NST), the biophysical profile (BPP), the contraction stress test (CST), ultrasound and amniotic fluid analysis, and fetal Doppler ultrasound employed in women with diabetes during pregnancy to identify fetuses at risk of adverse outcomes. The second aim of this review is to analyze the recommendations of international guidelines and clinical studies that evaluated the optimal timing for delivery in pregnant women with diabetes. By analyzing current clinical recommendations and contrasting them with recent research outcomes, we aim to provide a comprehensive and up-to-date view on optimizing antepartum care in this patient population.

## 2. Methods

Two authors searched for available information regarding GDM and PDM, assessing the main fetal surveillance tests currently used and the appropriate timing for resolving pregnancies complicated by DM.

To this end, a search in PubMed, MEDLINE, Google Scholar, and Scopus was performed to find relevant papers related to our objective, including the following MeSH terms: “gestational diabetes mellitus”, “diabetes mellitus”, “fetus”, “cardiotocography”, “umbilical artery”, “doppler ultrasound”, “prenatal ultrasonography”, “pregnancy”, “delivery”, “cesarean section”, “guideline”, “therapeutics”, and the following keywords: “pregestational diabetes mellitus”, “antepartum fetal surveillance”, “resolution of pregnancy”, “labor”, “biophysical profile”, “non-stress test”, and “treatment”. The search was performed between April and 1 August 2023.

The search was conducted using leading international diabetes guidelines including the National Institute for Health and Care Excellence (NICE), the American College of Obstetricians and Gynecologists (ACOG), the International Federation of Gynecology and Obstetrics (FIGO), the American Diabetes Association (ADA), the Society of Obstetricians and Gynaecologists of Canada (SOGC) and the Endocrine Society (ES).

Articles considered for inclusion in this review had to meet the following criteria: a focus on antepartum fetal surveillance and optimal timing of delivery in diabetic women, publications in peer-reviewed scientific journals, original studies, systematic reviews, meta-analyses, and clinical guidelines, with a publication date between 2012 and 1 August 2023 to ensure contemporary relevance.

The selection of articles was conducted in two stages. In the first stage, two independent researchers (ABP, ERM) reviewed titles and abstracts blinded to the authorship, author affiliations, and study results to determine their potential relevance. In the second stage, preselected articles were read in full to ensure compliance with the inclusion criteria and to extract relevant information. Studies not published within the established dates, in indexed journals, or whose full text was unavailable were excluded from this review.

Data extraction was performed using a standardized form, including study characteristics (author, year, country, study design), details of antepartum fetal surveillance methods used in diabetic women, findings related to maternal and neonatal outcomes, and recommendations regarding optimal delivery timing in this population. The results obtained from the selected articles were analyzed and organized for presentation in relevant sections of the article. The most relevant findings were highlighted, and connections were established between different fetal surveillance approaches and recommendations regarding delivery timing.

## 3. Results

We identified a total of eight international guidelines focused on diabetes and pregnancy, and AFS, as well as the optimal timing of delivery (ADA [1], three ACOG guidelines [3,20,30], NICE [11], FIGO [13], SOGC [12], and ES [14]). Additionally, considering the fetal surveillance tests, after removing duplicates, 17 observational and clinical studies were evaluated, which allowed for an objective recommendation to be provided as a complement to the international guidelines.

Our review identified a variety of AFS methods used in women with diabetes during pregnancy. These methods included monitoring fetal heart rate, biophysical profiles, and Doppler ultrasound of uterine and umbilical blood vessels. Table 3 and Table 4 show a summary of the principal recommendations of the international guidelines.

### 3.1. Non-Stress Test

The non-stress test (NST) has been used in various hospital settings as an initial assessment parameter in women with high-risk pregnancies, allowing the evaluation of fetal cardiac autonomic function [25].

Routine prenatal NST was implemented decades ago in clinical practice for pregnant women with DM without much available evidence to support it. As a result, the optimal timing and frequency of NST in women with diabetes have been subject to ongoing debate, leading to a need for more consensus regarding the most appropriate testing regimen [3,20,29]. According to the ACOG, NST is recommended for patients with PDM and GDM with poor glycemic control [3,29].

The ACOG and SOGC guidelines recommended weekly evaluation with NST among women with GDM and PDM starting at 36 weeks of pregnancy [3,12].

The NICE guidelines do not recommend the routine monitoring of fetal well-being with NST in GDM and in women with PDM starting at 38 weeks of gestation unless there is a risk of fetal growth restriction [11].

### 3.2. Biophysical Profile

The biophysical profile (BPP) is an adjunct test to identify fetal hypoxemia or acidemia constructed from data obtained from two sources: (1) the results of observations obtained via a dynamic ultrasound method of the presence or absence of fetal breathing movements, gross body movements, tone, and amniotic fluid volume; and (2) the results of the fetal heart rate responses to fetal movements as recorded using an NST [31,32]. Two points are assigned for each component meeting the specified criteria, and the final score is classified as normal (total score of 10; all components meet criteria or total score of 8; with a maximum vertical amniotic fluid pocket (MVP) > 2 cm), equivocal (total score of 8 but MVP below 2 cm or total score of 6 with an MVP > 2 cm), abnormal (total score of 6 with an MVP below 2 cm or total score of 4), or very abnormal (total score ≤ 2) [25,28,31,32].

The modified BPP evaluates the fetal heart rate reactivity and amniotic fluid by means of ultrasound measurement of the four-quadrant amniotic fluid index or MVP; the modified BPP is normal when the NST and amniotic fluid are normal. A nonreactive NST or an abnormal MVP requires further evaluation, typically by a full BPP [32].

In women with GDM and PDM, the ACOG recommends weekly BPP initiating at 32 weeks of gestation. However, testing at earlier gestational ages may be justified in some pregnancies complicated by additional high-risk conditions. If abnormal results are obtained in the NST, fetal surveillance tests can be repeated twice a week [3,29,30].

On the other hand, regarding GDM and PDM, FIGO and ADA guidelines recommend performing BPP at 32 weeks of gestation, with weekly follow-ups in the case of abnormalities [13,21,22,23,29].

In the presence of GDM, the NICE guidelines do not recommend the routine use of the biophysical profile. However, in the presence of PDM, they recommend that at 32 weeks of pregnancy, it can be started earlier in cases with a high risk of fetal demise [1,8,11].

### 3.3. Contraction Stress Test (CST)

The oxytocin tolerance test, or contraction stress test (CST), is based on the fetal heart rate (FHR)’s response to uterine contractions induced using oxytocin or direct stimulation on the mother’s nipples. It is based on the premise that uterine contractions will transiently affect fetal oxygenation [29]. The FHR is measured simultaneously with uterine contractions using an external fetal monitor. A good contraction pattern is characterized by at least three persistent contractions, each lasting 40 s within 10 min [10,29].

The CST allows for the simultaneous assessment of FHR and uterine contractions. It is primarily used when other FSTs (NST, BPP, ultrasound) yield positive or inconclusive results. The CST evaluation usually begins between 32 and 34 weeks of pregnancy. A regular or negative result indicates that FHR does not decrease following a uterine contraction [10,29].

However, because in clinical practice, there is less reliance on this FST, currently, there needs to be a consensus on the initiation, frequency, and follow-up of performing CST in patients with GDM [3,10,11,13,22,23].

### 3.4. Ultrasound and Amniotic Fluid Analysis

The NICE guidelines and SOGC recommend using fetal ultrasound starting at 28 weeks, with follow-up at 32, 38, and 39 weeks of gestation, analyzing fetal growth and assessing amniotic fluid [11,12]. Likewise, the ACOG guidelines recommend an ultrasound evaluation of amniotic fluid volume and fetal growth at four-week intervals starting from diagnosis (usually at 28 weeks of pregnancy) until resolution [3,10].

Similarly, weekly evaluation of fetal well-being (fetal kick counts, non-stress test, and biophysical profile) is suggested from 36 weeks of gestation (SOGC) or during weeks 38 and 39 (NICE) due to the significant increase in perinatal mortality associated with GDM [11,12].

On the other hand, FIGO suggests performing fetal ultrasound every two to four weeks from the diagnosis of GDM until the resolution of pregnancy, assessing fetal growth and development [13]. However, the American Diabetes Association (ADA) and the Endocrine Society (ES) do not establish a fixed criterion for the initiation and ultrasound follow-up in patients with GDM [1,8,14].

Moreover, a 2022 systematic review conducted by David and Spencer underscores fetal ultrasound as a pivotal tool for gauging fetal weight, estimating body composition, and evaluating potential complications and alterations that may arise in the offspring of mothers with high-risk pregnancies (including conditions like IUGR and fetal macrosomia) [33].

#### Fetal Doppler Ultrasound

The study of maternal and fetal blood flow has allowed for detecting anomalies throughout pregnancy, particularly abnormalities in growth (IUGR and macrosomia) and placental insufficiencies [21,22,34]. Doppler ultrasound of the umbilical artery is a valuable prenatal test that can help to ensure fetal well-being and prompt intervention when necessary [22,29,34]. Doppler ultrasound of the uterine artery during the first and early second trimester can be employed to predict pregnancies at risk of adverse outcomes, particularly early-onset preeclampsia and fetal growth restriction (FGR). Both conditions manifest through placental insufficiency, wherein the development of uteroplacental circulation is diminished due to the inadequate trophoblast transformation of spiral arteries into high-flow, low-resistance vessels [33,34]. It is recommended that high-risk pregnancies be monitored with Doppler ultrasound studies of the umbilical artery. Doppler ultrasound evaluation of placental circulation is crucial in detecting impaired placentation and fetal growth complications. Its purpose is to predict fetal acidemia, allowing for timely delivery before irreversible damage to vital organs occurs [13,29,30]. According to a systematic review, Doppler ultrasound velocimetry of the umbilical and fetal arteries in high-risk pregnancies, when followed by appropriate intervention, can reduce perinatal mortality rates by 29% and fetal death rates by 35%, respectively [22].

Considering the opinions provided previously, weekly or bi−weekly Doppler ultrasound assessment of the umbilical artery can help to prevent admission to the neonatal intensive care unit (NICU) and various complications in the early postpartum period [10,34].

### 3.5. Antepartum Fetal Surveillance Tests and Large for Gestational Age Neonates

Most international guidelines focused on small for gestational age (SGA) and LGA fetuses primarily address the risks associated with SGA fetuses regarding the likelihood of mortality or adverse effects on the fetus and neonates. However, the effects on LGA fetuses are less extensively discussed. In a retrospective cohort study conducted by Carter et al. [35], the incidence of stillbirth in pregnancies with LGA fetuses was significantly higher than in pregnancies with appropriate weight for gestational age (AGA) ≥ 36 weeks of gestation (26/10,000 vs. 7/10,000; adjusted odds ratio, 3.10; 95% CI, 1.68–5.70), and five times the incidence of stillbirths among LGA fetuses of pregnancies with diabetes ≥ 36 weeks of gestation compared to AGA pregnancies with diabetes adjusted odds ratio 5.11 (95% CI 1.44–18.14). This study demonstrated that stillbirth was associated with both growth restriction and excessive fetal growth, with a stronger association in more severe LGA cases (>95th percentile) [35]. The conclusion drawn was that LGA fetuses may benefit from antenatal testing starting at 36 weeks as a strategy to reduce the risk of stillbirth [35]. Similarly, in a national population-based retrospective cohort study by McElwee et al. [36], pregnancies complicated by both GDM and PDM and affected by pathologic fetal growth have an increased risk of stillbirth as gestational age advances. The risk is notably higher with pregestational diabetes, particularly in cases involving LGA fetuses. The study found that pregnancies complicated by pregestational diabetes had a relative risk of stillbirth of 21.8 (95% CI 17.4–27.2) for LGA fetuses and 13.5 (95% CI 8.5–21.2) for SGA fetuses compared with GDM-related AGA pregnancies at 37 weeks of gestation [36]. Meanwhile, a study examining the risk of LGA (≥97th percentile) singleton births at early term, full term, and late term concerning maternal pre-pregnancy BMI status mediated through GDM that analyzed data from 3,229,783 singleton term births concluded that LGA births are becoming more common, and the prevalence of maternal obesity and GDM is increasing. However, the proportion mediated through GDM was relatively small [19].

### 3.6. Pregnancy Resolution

The ACOG guidelines state that women with GDM with reasonable glycemic control without other complications can be managed expectantly until term. In most cases, patients with good glycemic control receiving pharmacological therapy can expect the resolution of their pregnancy at 39 weeks [3]. Therefore, the delivery timing in women with GDM managed with diet and exercise alone should not be before 39 weeks of gestation unless otherwise indicated from an obstetric point of view. The resolution of pregnancy at 38 to 39 weeks is recommended for women who require medication and are in great glycemic control [3]. The ACOG recommends that women with GDM be counseled regarding the risks and benefits of a scheduled cesarean delivery when the estimated fetal weight by ultrasound is 4500 g or more. This guideline also suggests that pregnancies in women with poorly controlled GDM should be delivered at 37 to 38 weeks of gestation [3]. However, according to FIGO guidelines, elective cesarean section at 38–39 weeks should be considered when the estimated fetal weight is above 4000 g [13].

In general, the NICE, FIGO, and SOGC guidelines state that the delivery timing in GDM pregnancies should emphasize glycemic control and the presence of complications or comorbidities. According to NICE and FIGO, uncomplicated and well-controlled cases of GDM should be managed expectantly until 40.6 weeks of gestation, and labor induction should be offered if spontaneous labor has not occurred [11,12,13].

Due to the increased risk of neonatal hypoglycemia in cases of maternal hyperglycemia during labor, NICE and FIGO recommend monitoring blood glucose levels, which should be maintained between 72 and 126 mg/dL [11,13].

According to the FIGO guidelines, the management of labor and delivery in patients with GDM and PDM depends on obstetric conditions, the status of the fetus, and the mother’s health. In cases where macrosomia is suspected with a fetal weight exceeding 4000 g, a cesarean section may be recommended starting at 38 weeks of gestation. However, DMG alone is not an absolute indication for a cesarean section [13].

## 4. Discussion

### 4.1. Evidence-Based Proposal for Fetal Surveillance in Diabetic Pregnant Women

**Figure 1:**
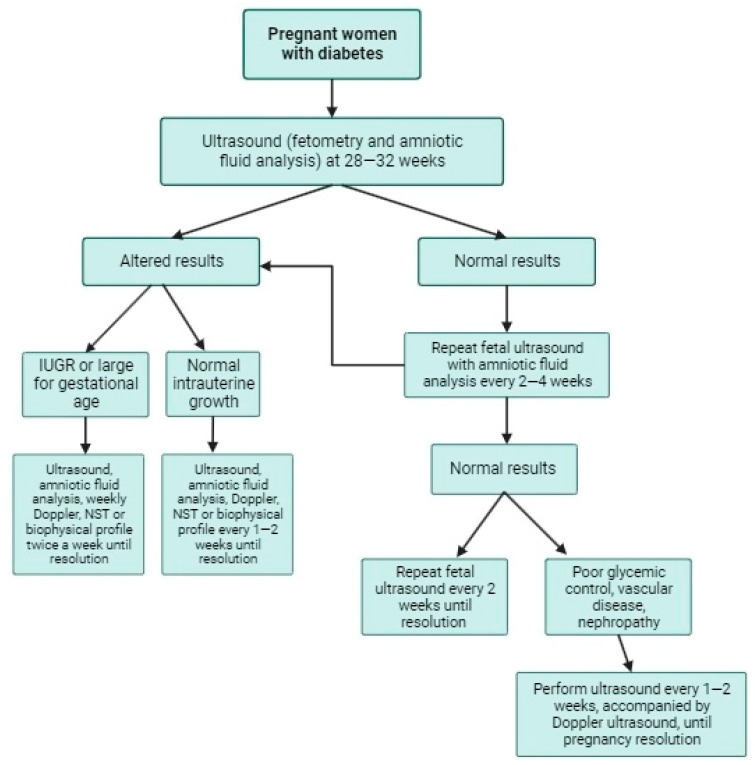
Recommendation in antepartum fetal surveillance tests in women with gestational diabetes mellitus and pregestational diabetes mellitus. IUGR = Intrauterine grow restriction.

This review evaluated the different international guidelines to find recommendations regarding starting and the type of AFS tests in women with DM. In general, the use of ultrasound and amniotic fluid analysis is recommended as the initial test, starting at 28–32 weeks of gestation, and followed by evaluations every two or four weeks. NST and BPP are recommended only in two international guidelines as optional AFS tests. The use of fetal Doppler ultrasound is recommended in women with DM and fetal complications such as IUGR [3,11,12,13,15,29,30]. Figure 1 shows an evidence-based proposal for fetal surveillance in diabetic pregnant women based on the present review.

In general, the various international guidelines highlight initiating fetal surveillance at 32 weeks of pregnancy. However, the specific factors determining its initiation and monitoring may vary among guidelines [3,11,12,13,15,29,30].

According to the ACOG, current studies have not shown a significant increase in fetal outcomes (stillbirths) in well-controlled GDM patients without pharmacological treatment in the absence of other comorbidities. Therefore, fetal surveillance may not be necessary [3]. In the case of GDM controlled with medication and without other comorbidities, prenatal fetal surveillance can be considered once or twice a week starting at 32 weeks. If GDM is poorly controlled, prenatal fetal surveillance can be considered twice a week, starting at 32 weeks [3,29,30]. Therefore, various factors, such as glycemic control and other aspects associated with a higher risk of adverse pregnancy outcomes, could be used to determine the frequency and timing of the AFS [3,11].

Pregnancy with GDM and PDM involves a risk for both the mother and the fetus, increasing the likelihood of fetal macrosomia, cesarean deliveries, low APGAR scores, fetal hypoxia, and neonatal asphyxia, among others. Therefore, the current maternal condition should be evaluated to determine the appropriate timing for the pregnancy resolution that provides the most significant benefit for the mother and her baby [37,38].

Considering this, we propose a comprehensive, individualized, and personalized assessment of each patient, focusing on their current health status and treatment modality. This approach will guide prenatal monitoring based on international guidelines.

Emphasizing the maternal–fetal benefits in preventing short and long-term adverse outcomes, the biophysical profile, the non-stress test, ultrasound, amniotic fluid analysis, and Doppler ultrasound should be considered (currently, the routine use of the contraction stress test is controversial) [34,37,38,39].

These results reflect the intricacy of antepartum fetal surveillance and the optimal timing of delivery in women with diabetes, emphasizing the importance of personalized and evidence-based care to enhance maternal and neonatal outcomes in this vulnerable population.

### 4.2. Optimal Timing for Delivery in Diabetic Women

The review evidences that determining the optimal timing for delivery in diabetic women involves a delicate balance between the inherent risks of diabetes and the benefits of extended fetal development. The optimal timing of delivery for women with DM is a complex issue for patients and care providers and depends on several individual and contextual factors. While there is consensus on the importance of avoiding preterm birth, the literature suggests that delaying delivery beyond a certain point could raise the risk of complications due to fetal size and impaired placental function [1,16]. Waiting for the pregnancy to reach full term is recommended in uncomplicated and well-controlled cases. A cesarean section may be considered in the presence of macrosomia or the absence of labor [3,11,13,15,29,30].

Unexplained stillbirths in maternal DM could be attributed to maternal hyperglycemia, fetal hyperinsulinemia, fetal hypoxia, acidemia, and myocardial dysfunction due to glycogen deposition [37]. The ACOG reaffirms that PDM increases the risk of fetal demise during pregnancy (RR of 2.50, 95% CI, (1.39–4.48)). Inadequate glycemic control with pharmacological treatment, the presence of polyhydramnios, fetal macrosomia, renal disease, IUGR, and concomitant gestational hypertension further increase the risk of fetal death [30]. Likewise, maternal hyperglycemia leads to fetal hyperglycemia and hyperinsulinemia, increasing the risk of fetal hypoxia. In addition to fetal hypoxia, abnormalities in cardiac structure and function (hypertrophic cardiomyopathy) and placental anomalies can contribute to the risk of neonatal complications among infants of diabetic mothers [33].

According to Ye et al. [23], in a systematic review and meta-analysis published in 2022, comparing patients with normal glucose tolerance in pregnancy and GDM, patients with GDM without insulin therapy had a higher odds ratio of cesarean section, preterm birth, low 1 min Apgar score, macrosomia, and LGA newborns. It was found that patients with GDM treated with insulin had a higher likelihood of LGA newborns, respiratory distress syndrome, neonatal jaundice, or admission to the neonatal intensive care unit [19]. Similarly, there is an increased likelihood of neonatal death (RR: 1.59) in the absence of insulin use. This is attributed to various lethal complications such as respiratory distress syndrome, neonatal hypoglycemia, and jaundice [23].

The rates of severe neonatal morbidity related to asphyxia (neonatal seizures and/or hypoxic-ischemic encephalopathy) were significantly higher between neonates of mothers with PDM than neonates of mothers without DM. In addition, maternal overweight and obesity were associated with a higher risk of low Apgar scores and severe neonatal morbidity related to asphyxia [33,37,40]. According to Ornoy et al., women with PDM type 1, PDM type 2, and GDM had higher rates of cesarean sections, instrumental delivery, low APGAR scores, seizures, and hypoxic-ischemic encephalopathy, in association with poor glycemic control [38]. Similarly, according to Metcalfe et al., in a population-based cohort study, women with diabetes and their infants face an elevated risk of adverse events attributable to excessive fetal growth. Their study suggests that earlier delivery when fetuses are smaller may help mitigate these risks [41].

**Figure 2:**
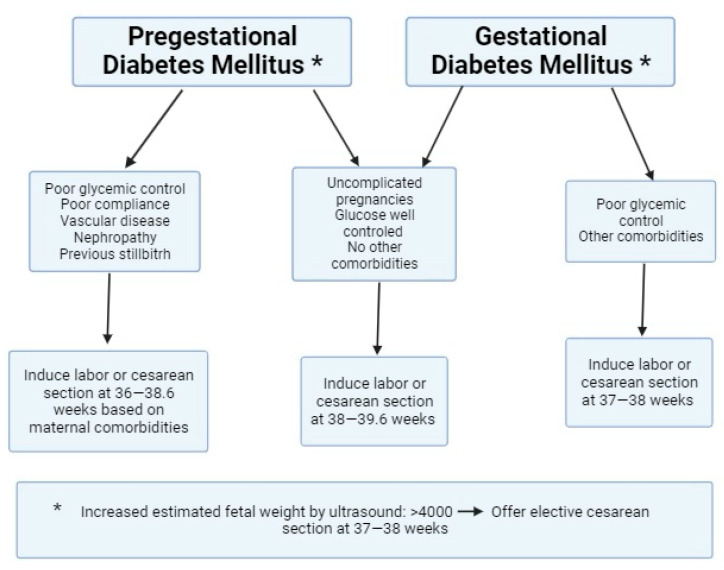
Algorithm: pregnancy resolution in women with diabetes mellitus [3,20,21].

This review highlights the necessity of addressing specific decision-making considerations related to delivery timing in diabetic women. These include the constant monitoring of blood glucose levels, assessing placental function, and adapting fetal surveillance strategies to optimize perinatal outcomes in this population. Figure 2 shows a proposed algorithm for pregnancy resolution in women with GDM and PDM based on the present review.

## 5. Conclusions

Diabetes in pregnancy represents a high risk for both the mother and the fetus. These aspects warrant close monitoring to reduce maternal–fetal complications. Assessing fetal well-being and detecting potential diabetes-related abnormalities are essential to the evaluation process. After analyzing the different international guidelines and relevant evidence on DM and pregnancy, the importance of proper AFS tests starting at 28–32 weeks of gestation and the use of ultrasound and amniotic fluid evaluation as initial test can be emphasized. Glycemic control, maternal comorbidities, and the medical management received play a crucial role in making decisions regarding the timing for the pregnancy resolution. There is a need for further studies focused on AFS tests and their relationship with the timing of pregnancy resolution, intending to identify and prevent the main complications that may arise during this period.

## Figures and Tables

**Table 1:** Risk factors for developing gestational diabetes mellitus [3,8].

Risk Factors		
Maternal age ≥ 25 years	Presence of twin pregnancy	Previous history of polycystic ovary syndrome
Belonging to the Hispanic, Asian, or African American ethnic group	Previous history of a child with birth weight > 4000 g or delivery of large for gestational age infant	Presence of glycosuria
Sedentary lifestyle	Excessive weight gain during pregnancy	Previous history of prediabetes (carbo-hydrate intolerance)
Family history of type 2 diabetes in a first-degree relative	Previous history of preeclampsia or hypertension during pregnancy	Presence of acanthosis nigricans
Hemoglobin A1C ≥ 5.7%, HDL cholesterol < 35 mg/dL, Triglycerides > 250 mg/dL	Previous history of gestational diabetes	Previous history of cardiovascular disease

**Table 2:** Recommendations of international guidelines for the diagnosis of GDM using the oral glucose tolerance test at 24–28 weeks of gestation.

Guideline	Glucose Load	Fasting (mg/dL)/mmol/L	1-h (mg/dL)/mmol/L	2-h (mg/dL)/mmol/L	3-h (mg/dL)/mmol/L
American Diabetes Association (2023) [1]	75 g *	≥92/5.1	≥180/10	≥153/8.5	
American Diabetes Association (2023) [1]	100 g **	≥95/5.3	≥180/10	≥155/8.6	≥140/7.8
National Institute for Health and Care Excellence (2020) [11]	75 g *	≥100/5.6	−	≥140/7.8	
Society of Obstetricians and Gynaecologists of Canada (2019) *** [12]	75 g **	≥95/5.3	≥190/10.6	≥162/8.9	
American College of Obstetricians and Gynecologists (2018) [3]	100 g **	≥95/5.3	≥180/10	≥155/8.6	≥140/7.8
Federation of Obstetrics and Gynecology (2015) [13]	75 g *	≥92/5.1	≥180/10	≥153/8.5	
Endocrine Society (2013) [14]	75 g *	≥92/5.1	≥180/10	≥153/8.5	
International Association of the Diabetes and Pregnancy Study Groups (2010) [15]	75 g *	≥92/5.1	≥180/10	≥153/8.5	

* One-step method: diagnosis of GDM with one or more altered values in oral glucose tolerance test. ** Two-step method: using a 50 g glucose challenge test, if the result is >140 mg/dL (7.8 mmol/L), perform the oral glucose tolerance test; diagnosis of GDM with two or more altered values; *** diagnosis of GDM with one or more altered values.

**Table 3:** Antepartum fetal surveillance tests in women with gestational diabetes.

	ACOG	NICE	FIGO	Other
Non-Stress Test	Weekly evaluation starting at 36 weeks of gestation.	No recommended	According to local protocol.	SOGC: Weekly evaluation starting at 36 weeks of gestation.
Biophysical Profile	Starting at 32 weeks of gestation. Follow-up twice a week in case of abnormalities.	No recommended	According to local protocol.	ADA: Starting at 32 weeks of gestation. Weekly follow-up.
Contraction Stress Test	Starting between 32 and 34 weeks of gestation. Controversial use.	Not discussed.	Not discussed.	Not discussed.
Ultrasound and Amniotic Fluid Analysis	Starting at 32 weeks of gestation. Growth ultrasound between 34 and 36 weeks of gestation.	Starting at 28 weeks of gestation, followed by evaluations at 32, 36 and 38–39 weeks of gestation.	Performing ultrasound every two to four weeks from the diagnosis until resolution of pregnancy.	SOGC: Starting with fetal ultrasound at 36 weeks of gestation, followed by weekly follow-up.
Fetal Doppler Ultrasound	Non-discussed. Used in high-risk pregnancies.	Used in pregnancies complicated with fetal growth restriction.	Non-discussed. Used in high-risk pregnancies.	Non-discussed. Used in high-risk pregnancies.

ACOG—American College of Obstetricians and Gynecologists; NICE—National Institute for Health and Care Excellence; FIGO—International Federation of Gynecology and Obstetrics; SOGC—Society of Obstetricians and Gynaecologists of Canada; ADA—American Diabetes Association; high-risk pregnancies—poor glycemic control, poor compliance, vascular disease, nephropathy, previous stillbirth.

**Table 4:** Antepartum fetal surveillance tests in women with pregestational diabetes.

	ACOG	NICE	FIGO	Other
Non-Stress Test	Weekly evaluation starting at 36 weeks of gestation.	Weekly at 38 weeks of gestation. Could start before in risk of fetal growth restriction	According to local protocol.	SOGC: Weekly evaluation starting at 36 weeks of gestation.
Biophysical Profile	Starting at 32 weeks of gestation. Follow-up twice a week in case of abnormalities.	Recommended after 32 weeks of pregnancy and can be performed earlier in cases of high risk of stillbirth	According to local protocol.	ADA: Starting at 32 weeks of gestation. Weekly follow-up.
Contraction Stress Test	Starting between 32 and 34 weeks of gestation. Controversial use.	Not discussed.	Not discussed.	Not discussed.
Ultrasound and Amniotic Fluid Analysis	Starting at 32 weeks of gestation. Growth ultrasound between 34 and 36 weeks of gestation. Amniotic fluid assessment starting at 32 weeks.	Starting at 28 weeks of gestation, followed by evaluations at 32, 36 and 38–39 weeks of gestation.	Performing ultrasound every two to four weeks until resolution of pregnancy.	SOGC: Starting with fetal ultrasound at 36 weeks of gestation, followed by weekly follow-up.
Fetal Doppler Ultrasound	Non-discussed. Used in high-risk pregnancies.	Used in pregnancies complicated with fetal growth restriction.	Non-discussed. Used in high-risk pregnancies.	Non-discussed. Used in high-risk pregnancies.

ACOG—American College of Obstetricians and Gynecologists; NICE—National Institute for Health and Care Excellence; FIGO—International Federation of Gynecology and Obstetrics; SOGC—Society of Obstetricians and Gynaecologists of Canada; ADA—American Diabetes Association; high-risk pregnancies—poor glycemic control, poor compliance, vascular disease, nephropathy, previous stillbirth.

## Data Availability

No new data were created or analyzed in this study. Data sharing is not applicable to this article.
